# Comprehensive cross-sectional and longitudinal comparison of sixteen markers of biological aging from the Berlin Aging Study II

**DOI:** 10.1038/s43856-025-01233-7

**Published:** 2026-03-27

**Authors:** Valentin Max Vetter, Johanna Drewelies, Jan Homann, Sandra Düzel, Laura Deecke, Philippe Jawinski, Simone Kühn, Elisa Kubala, Sebastian Markett, Michael Mülleder, Markus Ralser, Ulman Lindenberger, Christina M. Lill, Denis Gerstorf, Lars Bertram, Ilja Demuth

**Affiliations:** 1 Charité – Universitätsmedizin Berlin, corporate member of Freie Universität Berlin and Humboldt-Universität zu Berlin, Department of Endocrinology and Metabolic Diseases (including Division of Lipid Metabolism), Biology of Aging Working Group, Augustenburger Platz 1, Berlin, Germany; 2 Center for Environmental Neuroscience, Max Planck Institute for Human Development, Berlin, Germany; 3 Department of Psychology, Humboldt University Berlin, Berlin, Germany; 4 Institute of Epidemiology and Social Medicine, University of Münster, Münster, Germany; 5 Friede Springer Cardiovascular Prevention Center, Charité – Universitätsmedizin Berlin (CBF), Berlin, Germany; 6 Lübeck Interdisciplinary Platform for Genome Analytics (LIGA), University of Lübeck, Lübeck, Germany; 7 Center for Environmental Neuroscience, Max-Planck-Institut für Bildungsforschung, Berlin, Germany; 8 Max Planck UCL Centre for Computational Psychiatry and Ageing Research, Berlin, Germany, and, London, UK; 9 Core Facility High Throughput Mass Spectrometry, Charité - Universitätsmedizin Berlin, corporate member of Freie Universität Berlin and Humboldt-Universität zu Berlin, Berlin, Germany; 10 The Centre for Human Genetics, Nuffield Department of Medicine, University of Oxford, Oxford, UK; 11 Berlin Institute of Health (BIH) at Charité – Universitätsmedizin Berlin, Berlin, Germany; 12 Department of Biochemistry, Charité - Universitätsmedizin Berlin, corporate member of Freie Universität Berlin and Humboldt-Universität zu Berlin, Berlin, Germany; 13 Max Planck Institute for Molecular Genetics, Berlin, Germany; 14 Center for Lifespan Psychology, Max Planck Institute for Human Development, Berlin, Germany; 15 Aging Epidemiology Research Unit (AGE), School of Public Health, Imperial College London, London, UK; 16 Charité - Universitätsmedizin Berlin, Berlin Institute of Health Center for Regenerative Therapies (BCRT), Berlin, Germany

**Keywords:** Predictive markers, Predictive markers

## Abstract

**Background:**

The disproportionate increase in lifespan compared to healthspan over the past decades results in a growing proportion of life marked by diseases, even if incidence rates are falling in some cases. However, not everyone ages at the same pace and some people remain in good health and preserve physical and cognitive function into old age. To quantify inter-individual differences in the biological aging process, numerous indicators of biological age have been developed.

**Methods:**

In this study, we analyzed 16 measures of biological aging including epigenetic clocks, proteomics clock, telomere length, and SkinAge, laboratory composite markers (BioAge, Allostatic Load), psychological aging, and Brain Age. These age markers were evaluated cross-sectionally as well as longitudinally in the context of age-associated outcomes covering frailty, mobility, cognitive function, depressive symptoms, autonomy in daily life, nutrition, morbidity, and chronic disease in participants of the Berlin Aging Study II (BASE-II).

**Results:**

Here, we analyze longitudinal data from 1083 participants (mean age of 68.3 years at baseline, 52% women) with an average follow-up period of 7.4 years. Allostatic Load Index and DunedinPACE show the strongest and most consistent cross-sectional and longitudinal associations with age-associated phenotypes. Furthermore, both biomarkers individually increase the accuracy of a logistic regression model trained to predict incident cases of Metabolic Syndrome, high cardiovascular risk (Lifes’s Simple 7) as well as incident frailty (Fried’s frailty index) 7.4 years after baseline examination by up to 24 percentage points.

**Conclusions:**

Our findings support the previously shown distinction between indicators of aging and provide a comprehensive overview of their individual strengths and weaknesses in the context of wide variety of age-associated phenotypes.

## Plain language summary

People are living longer, but not everyone ages in the same way. Some people stay healthy well into old age, while others develop health problems earlier. To understand these differences and predict future health risks, various measures of biological aging have been developed, but comparisons of these measures in the same group of people are scarce. In this study, we compared 16 of these measures in more than 1000 older adults over seven years to see which aging markers best predict future health problems. We found that two measures, Allostatic Load and DunedinPACE, stood out as the most reliable for identifying people at risk of future problems such as frailty and cardiovascular risk. These findings could help physicians and scientists better understand aging and improve early detection of health problems in older adults.

## Abstract (web summary)

Vetter et al. investigate the cross-sectional and longitudinal relationship between 16 markers of ageing and 16 well-established age-associated phenotypes using data the Berlin Aging Study II. The results provide a comprehensive overview of the markers’ individual strengths and limitations and indicate their utility in incident case prediction.

## Introduction

Advances in healthcare, hygiene, lifestyle, and housing have led to an increase in the average lifespan in many countries over the past decades^1^. However, this expansion in lifespan was not matched by the increase in healthspan, the time spent before the onset of chronic disease or age-associated impairments^2–4^. At the same time, individual aging trajectories vary, and this heterogeneity increases with advancing age. While some individuals maintain good physical and mental health into old age, others show an early onset of chronic disease and impairment^5^. The geroscience hypothesis states that interventions targeting the biological process of aging may result in an increase of healthspan and prevent or at least postpone the onset of chronic disease^6^. Therefore, interventions that slow down or even reverse biological aging processes are of high individual but also societal interest^7^ but traditionally require long follow-up periods^3,8^. To test interventions in a cost- and time-effective manner, the validation of markers of aging that “either alone or in a composite predict biological age”^3^ is needed. Although extensive efforts have been made to identify and develop markers that can quantify inter-individual differences in biological age, no consensus has been reached^5,9–11^. This might be partly due to the differences in the conceptual meaning and the underlying framework of aging they aim to quantify and the methodological approach used. For example, aging clocks based on “-omics” data, like the proteomic clock or the first-generation epigenetic clocks, were trained to predict chronological age, and the difference between the predicted and the actual chronological age was shown to bear biological meaning^12^. In contrast, more recent versions of the epigenetic clocks are trained to predict age- and mortality-dependent biological changes or the so-called Pace of Aging^13,14^, and thus differ fundamentally in their underlying conceptualization. Other markers, such as BioAge^15^ and Allostatic Load^16^, are markers that aggregate laboratory measures associated with multiple organ systems into one composite biomarker of aging. Consistent with findings from our and other groups is that different indicators of aging only show a low to moderate association with each other^5,9,17–25^. This may result from the fact that the indicators were derived from different aging domains using varying methods. Whilst existing indicators of aging are individually well-evaluated, comparisons of different markers within the same study population and their relation to age-associated phenotypes are scarce^17,23^. Further, by focusing on selected health outcomes or high-risk populations, these studies limit our understanding of the broader predictive validity of aging markers in the general population.

To close this gap, we evaluate 16 markers of aging by investigating their relationship to a wide range of physical and mental age-associated health phenotypes in 1083 participants of the Berlin Aging Study II (BASE-II), based on both cross-sectional and longitudinal data. To assess their potential for clinical application, we additionally investigate the ability of each indicator to predict functional, physical and cognitive impairment after a mean follow-up period of 7.4 years. By doing so, we aim to provide a comprehensive overview of their individual strengths and weaknesses, in order to sharpen each biomarker’s individual profile, and contribute to a roadmap for their effective and targeted use in research and ultimately in clinical practice.

Our analyses reveal individual strengths and weaknesses of the investigated markers. DunedinPACE and Allostatic Load Index show the overall strongest and most consistent associations with the investigated outcomes. Both markers improve the discrimination of a basic clinical model developed to predict incident cases of Metabolic Syndrome, high cardiovascular risk (LS7), and frailty after a 7.4-year follow-up period by up to 24 percentage points.

## Methods

### Study population

BASE-II is an observational longitudinal study aiming at the identification of factors that predict and shape healthy aging trajectories^26^. Participants were recruited through the Max Planck Institute for Human Development’s participant pool in Berlin, as well as through advertisements in local newspapers and public transportation networks. As a convenience sample, baseline BASE-II participants were characterized by higher education and better self-reported health status than the general population of Berlin and Germany^26^. An above-average objective health status of BASE-II participants has been well documented, e.g., for diabetes mellitus type 2^27^, cardiovascular health^28^ and frailty^19^. Participants between the ages of 60 and 80 years (older cohort) and 20 and 35 years (younger cohort, not analyzed in this study) were eligible for recruitment. Men and women were recruited in equal numbers. The baseline examination (T0) of 1671 older participants (medical part) was conducted between 2009 and 2014^26^. A follow-up assessment (T1) as part of the GendAge study was conducted between 2018 and 2020^29^ on average 7.4 years after baseline (SD: 1.5 years, range: 3.9 to 10.4 years). Only participants who had provided information on outcomes of interest at baseline as well as at follow-up were included in the final sample (*n* = 1083, Supplementary Fig. 1), to allow comparison of the aging markers in the same cohort of individuals. Of the 588 participants who dropped out between baseline and follow-up, 126 were confirmed to have died. Reasons for dropout among the remaining 462 participants were not systematically evaluated. Differences in the variables analyzed in this study between the main study sample and participants who dropped out are small, and we do not expect that loss to follow-up substantially altered our findings (Supplementary Data 1).

All participants gave written informed consent. All assessments at baseline and follow-up were conducted in accordance with the Declaration of Helsinki and approved by the Ethics Committee of the Charité—Universitätsmedizin Berlin (approval numbers EA2/029/09, EA2/144/16, and EA2/224/21) and were registered in the German Clinical Trials Registry as DRKS00009277 (BASE-II) and DRKS00016157 (GendAge). The Ethics Committee of the Max Planck Institute for Human Development approved the procedure, and the Ethics Committee of the German Society for Psychology (DGPs) additionally approved of the MRI protocol. This manuscript was created in accordance with the STROBE guidelines^30^.

### Variables

The Horvath^31^, Hannum^32^, PhenoAge^33^, GrimAge^34^, and DunedinPACE^14^ epigenetic clock algorithms were applied to derive DNA methylation age (DNAmAge) from methylation data measured with the “Infinium MethylationEPIC”, version 1, array (Illumina, Inc., USA). Methylation data for the 7-CpG epigenetic clock^18^ was obtained through Single Nucleotide Primer Extension (SNuPE)^18,35^. DNAmAge acceleration (DNAmAA) was calculated as the unstandardized residuals of a leukocyte cell-count adjusted linear regression analysis of DNAmAge on chronological age. Proteomics Age was derived from 248 proteins measured by liquid chromatography-mass spectrometry (LC-MS). Proteomics Age acceleration (ProteomicsAA) was calculated similarly to the DNAmAA as residuals from a linear regression of Proteomics Age on chronological age. Telomere length was assessed through quantitative real-time PCR (rLTL^36^) as well as estimated by the algorithm proposed by Lu and colleagues^37^ from methylation data (DNAmTL). The average results across three raters evaluating the number of lentigines from photos of the participants’ skin were used to derive SkinAge^15^. BioAge is a composite score that aggregates 13 routine laboratory parameters that were identified for their association with mortality^15^. The Allostatic Load Index (ALI) was computed by awarding a point to every participant within the high-risk quartile of selected variables using the approach described by Seeman and colleagues^16^. It additionally incorporates information about intake of relevant medication to include successfully treated and thereby masked dysregulation in its calculation^38,39^. Subjective Felt Age (SFA) was calculated as proportional discrepancy score from self-reported “felt age” and chronological age^40^. Subjective Life Expectancy (SLE) is calculated as the difference between the age participants expect to live to and their chronological age at the time of assessment. Subjective Health Expectancy (SHE) is the difference between the age participants expect to remain healthy and their chronological age at the time of assessment. Both SLE and SHE were adjusted for chronological age^41^. In addition to these markers, BrainAge was available in a subgroup of *n* = 255 BASE-II participants who underwent Magnetic Resonance Imaging (MRI) in a 3-Tesla Siemens Magnetom Trio scanner. BrainAge was calculated using a model trained on participants from the UK Biobank^42^. Except for Proteomics Age, all other indicators of aging were individually investigated in BASE-II before^15,18,19,24,28,43–49^.

Outcome measures investigated in this study include two measures of frailty. The Fried Frailty Phenotype (Fried FI) includes information on unintended weight loss, exhaustion, weakness, slow walking speed, and low physical activity^50^. The SPRINT-BASEed frailty index (SP FI) used in this study is an adapted version^51^ of the deficit-based measure developed by Pajewsky and colleagues^52^. Finger Floor Distance (FFD) was measured in centimeters. The Tinetti Mobility Test assesses mobility and balance through a series of simple tasks that are rated by the test supervisor^53^. Mini Mental State Examination (MMSE) is a well-established interviewer-administered instrument that was used to assess cognitive impairment^54^. Additionally, cognitive performance, specifically processing speed, was measured by the Digit Symbol Substitution Test (DSST), for which participants were asked to match symbols to numbers according to a given key^55^. Depressive symptoms were assessed using the Center for Epidemiologic Studies Depression Scale (CES-D)^56^. Independence during everyday life and the ability to perform tasks without help was measured using the Activities of Daily Living questionnaire (ADL, “Barthel Index”)^57^. The nutritional status was assessed using results from a short questionnaire and the measured circumference of the upper arm and the calf (Mini Nutritional Assessment, MNA^58^). Type 2 Diabetes (T2D) was diagnosed based on the criteria defined by the American Diabetes Association (ADA) guidelines^59^. Diabetes-associated complications were quantified using the composite score developed by Young and colleagues^27,60^. The Morbidity Index (MI) was calculated to assess the overall morbidity burden of the BASE-II participants by adapting the approach first described by Charlson and colleagues^61^. The Systematic Coronary Risk Evaluation assessment (SCORE2^62^) and the SCORE2-OP (for participants >70 years, hereafter referred to as SCORE2^63^) were calculated according to the recommendations in the respective publications to assess the risk for cardiovascular events. An adapted version^28,64^ of the Life’s Simple 7 (LS7^65^) was calculated to quantify modifiable cardiovascular risk factors. Metabolic Syndrome (MetS) was diagnosed using the definition suggested by the American Heart Association/International Diabetes Federation/National Heart, Lung, and Blood Institute criteria 2009^66^. The outcome variables described above were chosen because they represent specific health aspects that we analyze as downstream effects of the biological aging process quantified by the investigated markers of aging. However, in other contexts, some of the outcome variables themselves could also be investigated as aging markers and vice versa.

Confounding variables included in the regression models were chronological age (years), sex, alcohol consumption (g/d), and nicotine consumption (packyears), which were assessed during 1:1 interviews with trained study personnel. Body Mass Index (BMI) was calculated as kg/m^2^ using measurements from an electronic measuring station (seca 763, SECA, Germany). Genetic ancestry was quantified using the first four components from a principal component analysis on genome-wide single-nucleotide polymorphism genotyping data^67^. Details on the variables investigated in this study are described in the Supplementary Material.

### Statistics and reproducibility

Statistical analyses and visualizations were conducted using R (version 4.3.2)^68^. Missing values were imputed by multiple imputation using the mice package (for detailed information, please see the Supplementary Methods). In this study, *n* = 1083 participants of BASE-II were analyzed. BrainAge was available in a subgroup of 255 BASE-II participants. To facilitate comparison with all other markers, a separate multiple imputation procedure including all other variables was applied in this subsample. Descriptive statistics of the first imputed dataset are presented in Tables 1 and 2. Descriptive statistics of the original (non-imputed) dataset are shown in Supplementary Data 2. Intercorrelation plots for markers of aging and outcome variables are presented in Supplementary Figs. 2 and 3. The change in outcome variables was assessed by subtracting the outcome value at T0 from the value documented for T1. The relationship between markers of aging and outcome variables was assessed by logistic and linear regression models. In line with previous publications in the field as well as recommendations for increased quality in biomarker validation, we report results from an unadjusted, a minimally adjusted (chronological age and sex), and fully adjusted model^69^. The longitudinal minimally and fully adjusted model is additionally adjusted for the outcome variables at T0. Since we were mainly interested in the raw predictive ability of the biomarkers (diagnostically as well as prognostically), we focus on the description of the unadjusted model in the main text. Statistical significance of the differences between the basic risk prediction models with and without the respective marker of aging was assessed using the *roc*.test function (pROC package). To adjust for multiple testing, a Bonferroni correction was applied to the main analyses. As cross-sectional and longitudinal analyses were performed, we set our level for statistical significance at α = 0.05/(2*16 markers*16 outcomes) = 0.0001. To facilitate comparison between markers and outcome measures on differing scales, all values were z-transformed using R’s *scale* function prior to the regression analyses, and standardized effect measures are reported. Dichotomization of continuously scaled variables used in the sensitivity analyses and the impairment prediction models was done using validated cut-off values if available (Supplementary Data 3). The MMSE and ADL were excluded from analyses that examined the dichotomized variables due to the very limited number of participants showing impairment according to these instruments. For the main analyses, sex-stratified subgroup analyses were performed as suggested by Moqri and colleagues^69^. No age-group stratified analyses are shown due to the already narrow age range in BASE-II^69^. Further information on the statistical analyses can be found in the Supplementary Material.

**Table 1:** Descriptive statistics of markers of aging at baseline in older participants of the BASE-II (*n* = 1083)

Variables	Mean (SD)	Min	Max
Chronological age	68.27 (3.49)	60.16	84.63
7-CpG DNAmAA	−0.02 (6.94)	−22.93	22.33
Horvath DNAmAA	0.08 (4.22)	−11.64	14.45
Hannum DNAmAA	−0.04 (3.43)	−10.9	19.15
PhenoAge DNAmAA	0.08 (4.65)	−16.88	14.28
GrimAge DNAmAA	0.07 (3.12)	−8.52	10.2
DunedinPACE	1.01 (0.11)	0.58	1.42
DNAmTL	6.97 (0.18)	6.26	7.44
rLTL	1.15 (0.23)	0.17	1.92
SkinAge	1.71 (0.90)	0	3
ProteomicsAA	−0.02 (1.34)	−4.18	5.11
BioAge	0.17 (5.91)	−16.37	20.21
Allostatic Load	3.98 (2.40)	0	12
Subj. age^a^	0.09 (0.08)	−0.13	0.41
Subj. life expectancy	15.89 (7.68)	0	47
Subj. health expectancy	11.47 (7.06)	0	46
BrainAge^b^	−0.06 (3.23)	−9.03	8.03

^a^Subj. age = relative subjective age adjusted by chronological age.

^b^BrainAge was available in a subgroup of 255 participants.

**Table 2:** Descriptive statistics of outcome variables at baseline (T0) and follow-up (T1) examination (*n* = 1083)

	T0				T1						
Variable	Mean, *n*	SD, %	Min	Max	Mean, *n*	SD, %	Min	Max	Mean Diff.	SMD	*p*-value
Chronological age	68.272	3.489	60.2	84.6	75.62	3.768	64.9	94.1	7.348	2.024	<2.2e-16
Fried frailty	0.364	0.61	0	3	0.763	0.878	0	4	0.399	0.528	<2.2e-16
Frailty SPRINT-BASEed	0.132	0.063	0.008	0.44	0.16	0.075	0	0.523	0.028	0.411	<2.2e-16
Finger floor distance	9.746	11.839	0	52	10.576	12.673	0	159	0.829	0.068	0.005
Tinetti score	27.629	1.691	0	28	26.572	2.668	7	28	−1.056	−0.473	<2.2e-16
Falls (past 12 months)	336	0.31			295	0.272					0.043
MMSE	28.59	1.335	22	30	28.578	1.524	18	30	−0.012	−0.008	0.827
DSST	45.001	8.301	5	74	40.801	8.674	2	73	−4.2	−0.495	<2.2e-16
CES-D	7.285	6.613	0	36	7.17	6.165	0	36	−0.115	−0.018	0.526
ADL	99.003	3.72	5	100	98.698	4.086	15	100	−0.305	−0.078	0.046
MNA	27.383	1.756	19	30	26.595	2.158	16	30	−0.788	−0.401	<2.2e-16
DCSI	0.777	1.163	0	7	1.264	1.486	0	8	0.488	0.365	<2.2e-16
Morbidity index	0.975	1.208	0	7	1.432	1.542	0	9	0.457	0.33	<2.2e-16
SCORE2	10.809	4.222	4	32	16.073	6.16	5	47	5.264	0.997	<2.2e-16
LS7	8.609	1.978	2	14	8.44	1.86	3	14	−0.17	−0.088	0.001
T2D (diagnosed)	127	0.117			185	0.171					1.874e-09
Metabolic syndrome (diagnosed)	388	0.358			491	0.453					5.018e-11

Two-sided paired *t*-test and McNemar’s test were used to assess the statistical significance of differences between timepoints.

*SMD* Standardized Mean Difference, *LS7* Life’s Simple Seven, *MMSE* mini mental state examination, *SCORE2* systematic coronary risk evaluation 2, *DCSI* diabetes complications severity index, *CES-D* Center for Epidemiologic Studies Depression Scale, *MNA* mini nutritional assessment, *DSST* digit symbol substitution test, *Tinetti* Tinetti test, *T2DM* type 2 diabetes mellitus.

## Results

### Participants

In this study, 1083 BASE-II participants who provided information at baseline and follow-up on average 7.4 years later were analyzed with respect to cross-sectional and longitudinal associations of 16 markers of aging (Supplementary Fig. 4) with a range of age-associated outcomes. Mean chronological age at baseline was 68.3 years (SD: 3.5 years), and 52% were women (Table 1, Supplementary Data 1). Frequency of impairment in the analyzed variables is shown in Supplementary Data 4. All age-associated outcome variables differed statistically significantly between examinations with the exception of FFD, Falls, MMSE, ADL, CES-D, and LS7 (Table 2). As described in BASE-II before^24^, correlations between markers of aging derived from different data sources and domains were moderate to low with r ≤ |0.31| while higher correlations were found between markers which were calculated based on the same data (e.g., epigenetic clocks, r ≤ 0.49) or which were closely related to each other (SHE and SLE, r = 0.73, Supplementary Fig. 2).

### Cross-sectional association between markers of aging and age-associated outcomes at baseline

**Fig. 1:**
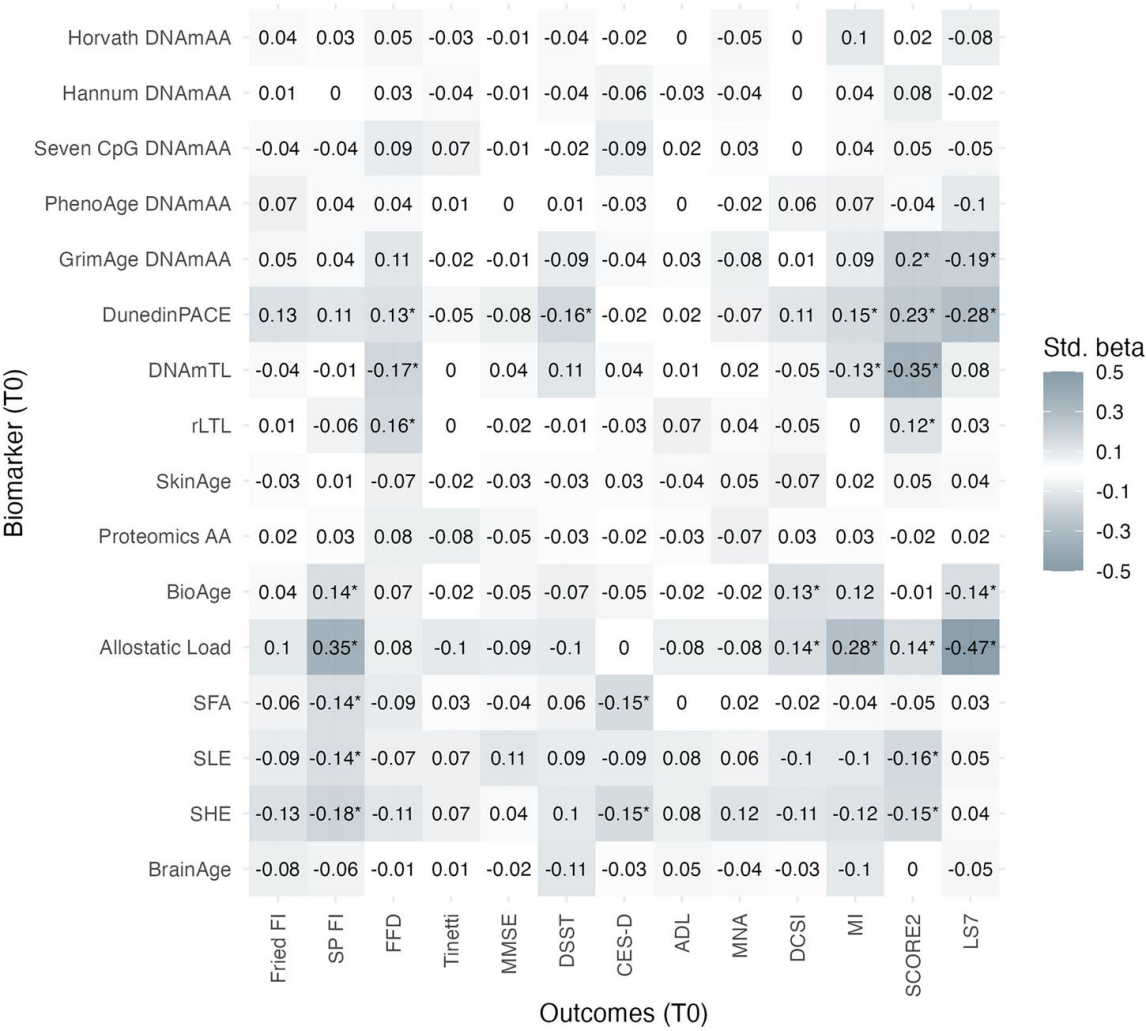
Standardized regression coefficients of unadjusted linear regression analyses of outcome variables on markers of aging in 1083 participants from the BASE-II at baseline. BrainAge was available in a subgroup of *n* = 255 participants. *Bonferroni-corrected statistical significance (*p* < 0.0001). LS7 Life’s Simple Seven, SCORE2 systematic coronary risk evaluation 2, MI Charlson’s morbidity index, DCSI diabetes complications severity index, MNA mini nutritional assessment, DSST digit symbol substitution test, Tinetti Tinetti test, FFD finger floor distance, SP FI SPRINT-BASEed frailty phenotype, Fried FI Fried’s frailty phenotype, MetS metabolic syndrome, T2DM type 2 diabetes mellitus.

Cross-sectional associations of markers of aging with continuously and categorically scaled age-associated outcomes at baseline were investigated using linear and logistic regression models. No statistically significant association (Bonferroni adjusted significance level defined at α = 0.0001) with the outcome variables was found for the first-generation epigenetic clocks, PhenoAge DNAmAA, SkinAge, ProteomicsAA, and BrainAge. In contrast, GrimAge DNAmAA, DunedinPACE and both laboratory composite markers (BioAge, ALI) were statistically significantly associated with at least one of the two cardiovascular health scores (LS7, SCORE2, all *p* > 0.000018). In addition, ALI and DunedinPACE were associated with disease-related outcomes like MI (*p* = 7.3 × 10^−^^6^), diagnosed T2D (*p* = 5.7 × 10^−^^6^), and MetS (*p* = 1.1 × 10^−^^10^, Supplementary Data 5). After correction for multiple testing, both TL measures were statistically significantly associated with FFD and SCORE2, and all three psychological aging markers (SFA, SLE, SHE) were statistically significantly associated with SPRINT-BASEed FI. Overall, the strongest and most robust results after covariate adjustment were found for ALI and DunedinPACE. All results from the unadjusted cross-sectional linear and logistic regression analyses, as well as minimally (age, sex) and fully adjusted models (age, sex, genetic ancestry, alcohol intake, smoking, BMI) are displayed in Fig. 1, Supplementary Fig. 5, and Supplementary Data 5 and 6.

As a sensitivity analysis, we report on the results of logistic regression models of dichotomized continuously scaled variables on the markers of aging (Supplementary Data 6) and sex-stratified subgroup analyses (Supplementary Data 5 and 6). All analyses were repeated in the subsample of participants for which BrainAge was available (Supplementary Data 7 and 8) to allow direct comparison of the results of BrainAge with the other markers in the same individuals.

### Longitudinal associations between markers of aging at baseline and age-associated outcomes after 7.4 years of follow-up

**Fig. 2:**
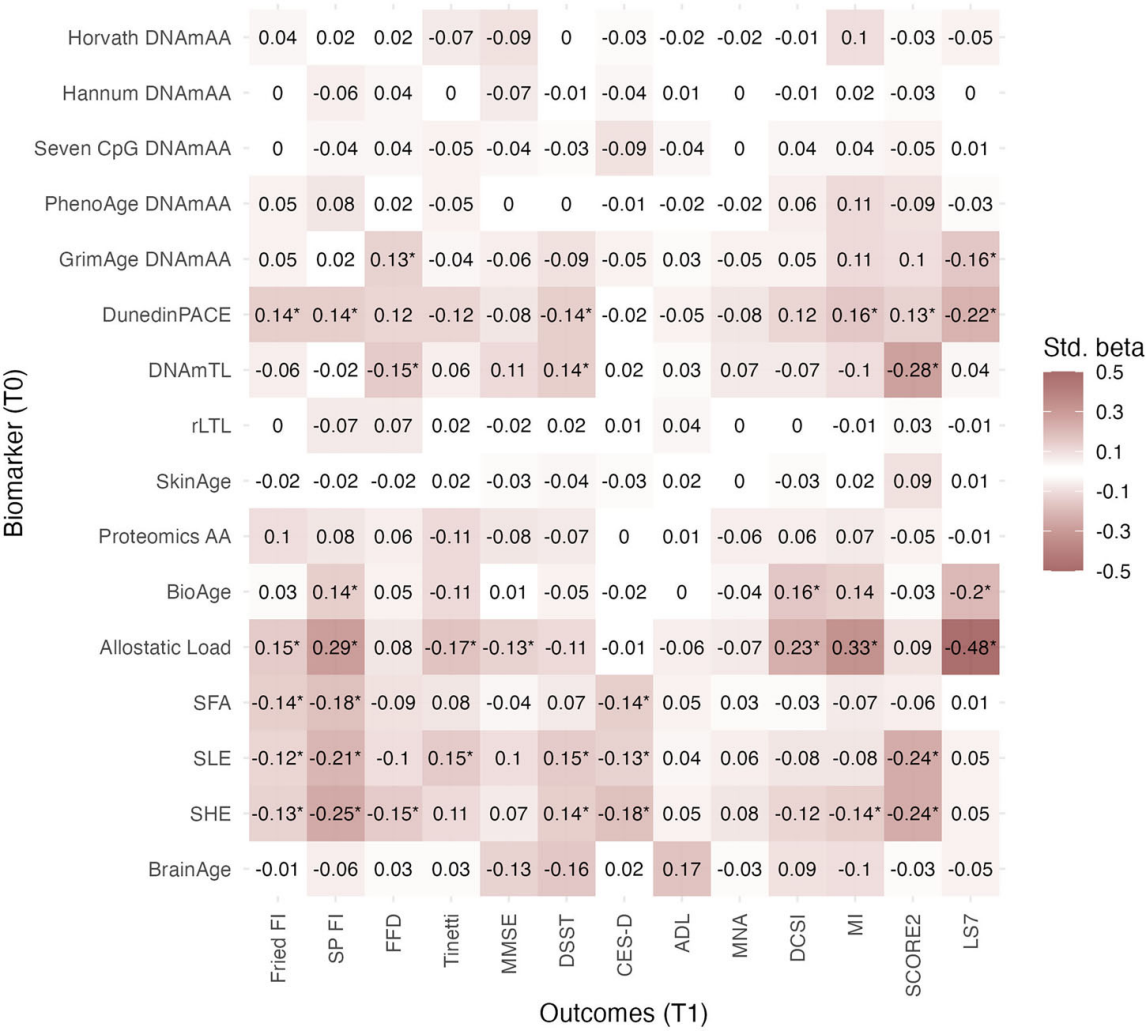
Standardized regression coefficients of unadjusted longitudinal linear regression analyses of outcome variables at T1 on markers of aging at T0 in 1083 participants from the BASE-II. BrainAge was available in a subgroup of *n* = 255 participants. *Bonferroni-corrected statistical significance (*p* < 0.0001). LS7 Life’s Simple Seven, SCORE2 systematic coronary risk evaluation 2, MI Charlson’s morbidity index, DCSI diabetes complications severity index, MNA mini nutritional assessment, DSST digit symbol substitution test, Tinetti Tinetti test, FFD finger floor distance, SP FI SPRINT-BASEed frailty phenotype, Fried FI Fried’s frailty phenotype, MetS metabolic syndrome, T2DM type 2 diabetes mellitus.

To explore the markers’ longitudinal association with the investigated outcomes, we performed regression analyses of outcomes measured at follow-up on markers assessed at baseline. Similarly to the cross-sectional analyses, no statistically significant associations with age-associated outcomes were found for measures derived from first-generation epigenetic clocks, PhenoAge, SkinAge, ProteomicsAA, and BrainAge. As already observed in the cross-sectional analyses, after correction for multiple testing, the strongest and most consistent associations were found for DunedinPACE and the ALI. Interestingly, all three psychological aging markers were associated with Fried’s frailty phenotype in addition to the association with the SPRINT-BASEed frailty index that was already observed in the cross-sectional analyses at baseline (Fig. 2, Supplementary Data 9). The longitudinal results of the dichotomized as well as of the binary outcome variables can be found in Supplementary Data 10 and Supplementary Fig. 6. The results from the analyses of the subgroup of participants who provided BrainAge data can be found in Supplementary Data 11 and 12. Additionally, we investigated the change in the continuously scaled outcome variables by regressing the change in the outcome variables on the markers of aging at baseline, which can be found in Supplementary Data 13. Similar to the other longitudinal linear regression models, ALI showed the most frequent and consistent statistically significant associations across outcomes and models.

### Prediction of incident cases in outcome variables over a 7.4-year follow-up period

To promote translation into clinical practice and to evaluate the markers in a possible use-case scenario, we evaluated the markers’ ability to predict the incidence of impairment in the analyzed assessments, as well as incident cases of the investigated diseases and frailty over the average follow-up time of 7.4 years. Each marker-outcome-combination was evaluated in two scenarios. First, we simulated the case in which no other clinical information is available and assessed the markers raw predictive value. In a second step, we investigated the additional value that each marker adds to a basic prediction model.

**Fig. 3:**
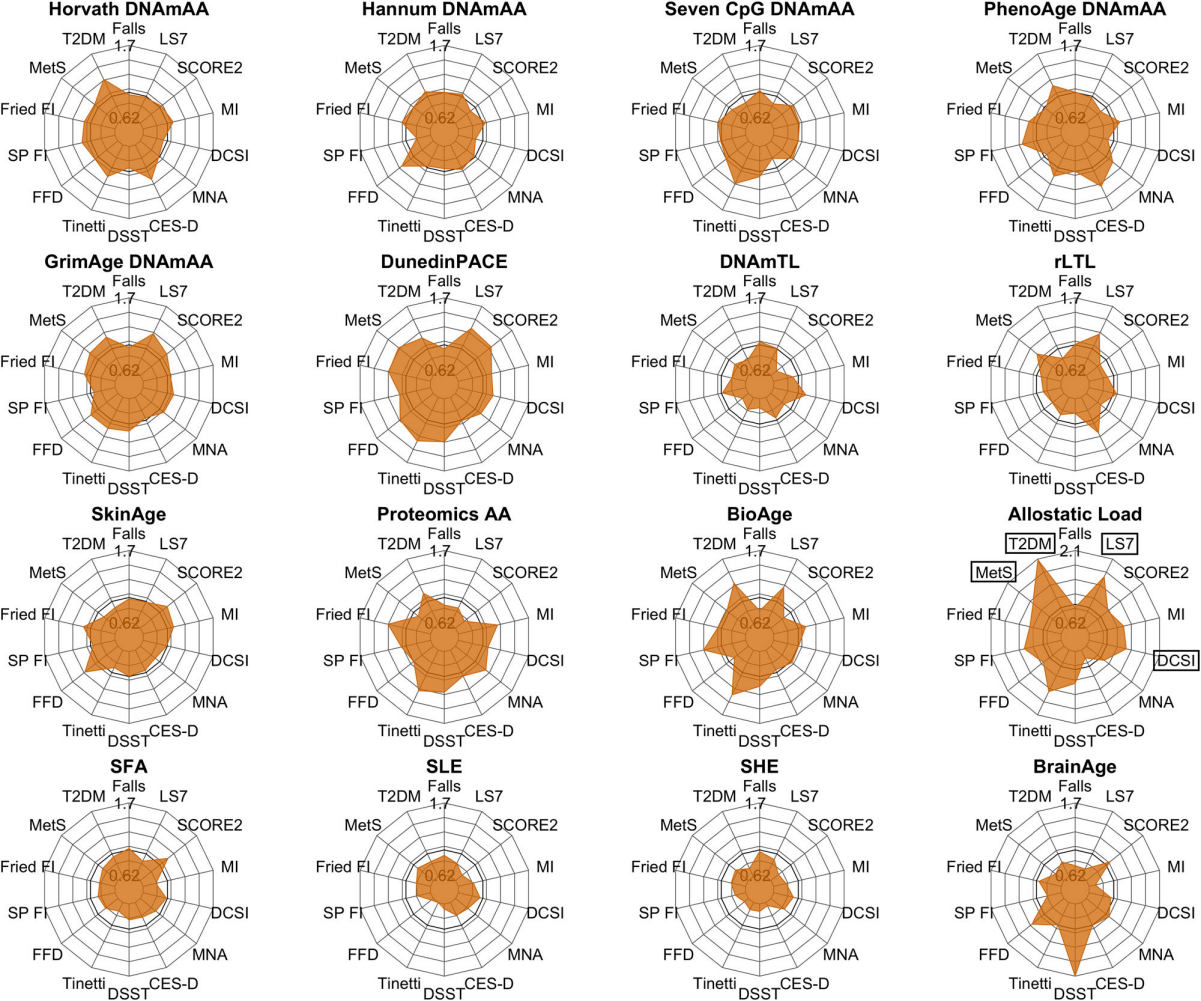
Radar plots showing the results of unadjusted logistic regression analyses of incident cases of analyzed outcome variables at T1 on markers at T0. Participants with prevalent cases at T0 were excluded from this analysis. Due to the multiple imputation procedure, the sample sizes between each imputed dataset differ slightly. The range of sample sizes is indicated in Supplementary Data 18. Due to very strong effect sizes observed for Allostatic Load, the scale of this plot was adjusted to increase readability of the other plots. Therefore, the individual axis limits are displayed for each plot individually. Variable names of associations that were statistically significant after Bonferroni correction for multiple testing (*p* < 0.0001) are displayed within a black box. LS7 Life’s Simple Seven, SCORE2 systematic coronary risk evaluation 2, MI Charlson’s morbidity index, DCSI diabetes complications severity index, MNA mini nutritional assessment, DSST digit symbol substitution test, Tinetti Tinetti test, FFD finger floor distance, SP FI SPRINT-BASEed frailty phenotype, Fried FI Fried’s frailty phenotype, MetS metabolic syndrome, T2DM type 2 diabetes mellitus.

**Fig. 4:**
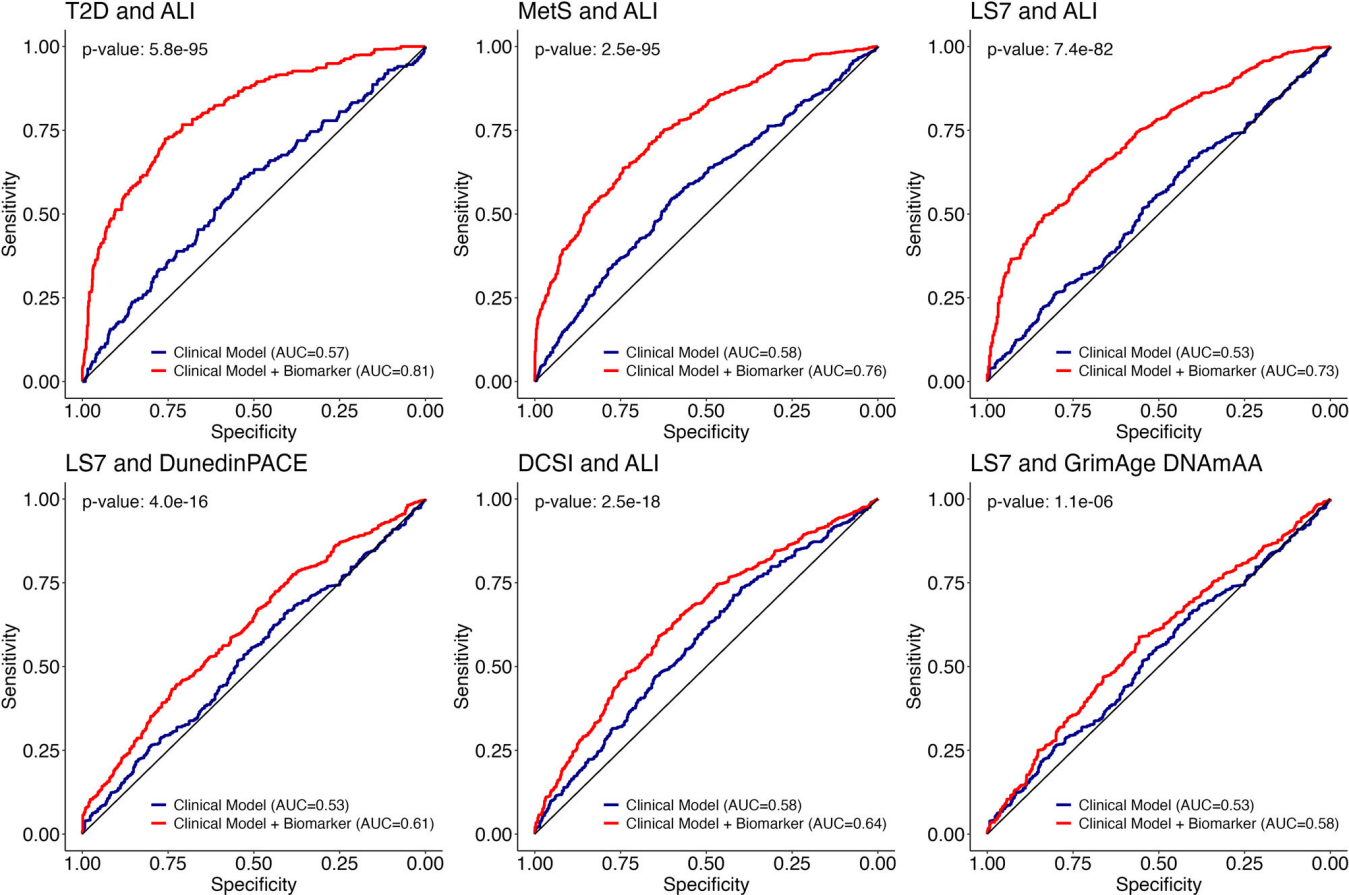
ROC curves illustrating the sensitivity and specificity of selected logistic regression models predicting impairment in age-associated phenotypes on average 7.4 years after marker assessment. The predictive performance of a basic clinical model including age and sex (blue) is shown compared to a prediction model that is extended by the respective markers of aging (red). The marker-outcome combinations presented here were selected to highlight the most compelling results from the three most promising markers (ALI, DunedinPACE, and GrimAge). A complete list of all AUC values can be found in Supplementary Data 18. *P*-values for the difference between prediction models were calculated using the approach described by DeLong^72^ as part of the roc.test function (pROC package). After Bonferroni correction, the level of statistical significance was defined at *p* < 0.0001.

Of all analyzed biomarkers, Allostatic Load was the only one to be statistically significantly (after multiple testing correction) associated with incident impairment at follow-up (Fig. 3). Specifically, higher Allostatic Load was associated with increased odds for a diagnosis of T2D and MetS and its addition to the basic prediction model improved the AUC of the model by 24 and 18 percentage points (DeLong’s test, *p* < 0.00001, Fig. 4). Additionally, an increase in one SD of Allostatic Load at baseline increased the odds for the incidence of at least one diabetes-associated complication at T1 (DCSI) 1.4-times (*p* < 0.0001) and the inclusion of Allostatic Load to a basic prediction model increased the AUC by 7 percentage points (*p* < 0.00001). Odds for an incident classification as “average” or “inadequate” based on the LS7 criteria were 1.7-fold for every 1-SD increase in Allostatic Load (*p* = 0.00002, Fig. 4) at baseline and it increased the accuracy of the basic prediction model by 20 percentage points (*p* < 0.00001, Fig. 4). Interestingly, while Allostatic Load was not statistically significantly associated with the outcome in prediction models for Fried’s frailty index and MI, its inclusion still improved the respective prediction models statistically significantly by 4 and 10 percentage points (*p* < 0.00003), respectively. To investigate the robustness of this association, we conducted a series of sensitivity analyses with modified versions of the ALI. If the analyzed outcome or variables used in its definition was also part of the ALI, as is the case for MetS, T2D, LS7, SCORE2, the respective variables were individually excluded from the calculation of the modified ALI. As expected, effect sizes of the association were stronger for the original ALI in comparison to the modified ALI scores (Supplementary Data 14–16). However, in almost all cases, the associations remained statistically significant, which increases confidence that ALI is a robust marker for biological age. Similarly, DunedinPACE did not show individual statistically significant associations in the models, but its addition to the basic prediction model increased the AUC of models predicting diagnosis of MetS and impairment in Fried’s frailty index, and LS7 by 5, 4, and 8 percentage points (*p* < 0.00006, Fig. 4). In case of LS7, GrimAge DNAmAA improved the prediction model by 5 percentage points (*p* < 0.0001, Fig. 4). As a sensitivity analysis, we re-calculated all prediction models with GrimAge2 instead of GrimAge and found no or small differences (≤1.6 percentage points in the AUC) between the two versions (Supplementary Data 17). A very high AUC (AUC = 0.935) was observed for the clinical prediction model with respect to identifying incident high cardiovascular risk defined by the SCORE2 algorithm. This prediction was significantly improved by 1 percentage point after adding TL measured using qPCR (*p* < 0.00003). The complete results for all marker-outcome combinations are shown in Fig. 3 and Supplementary Data 18.

## Discussion

In this study, we investigated 16 markers of aging in 1083 older participants from the Berlin Aging Study II (BASE-II). All markers were cross-sectionally and longitudinally analyzed in the context of a wide range of age-associated outcome variables representing different aspects of aging, including frailty, mobility, cognitive function, depressive symptoms, autonomy, nutrition, overall health, and chronic disease. Additionally, we investigated the markers’ ability to predict incident impairment in the age-associated outcomes over the average follow-up period of 7.4 years.

Our analyses showed that GrimAge DNAmAA and DunedinPACE performed best with respect to their association with cardiovascular health and cognitive capacity. Allostatic Load seems to represent frailty and overall morbidity-associated variables (MI, T2D and MetS). Longitudinally, the subjective psychological markers were associated with depressive symptoms (CES-D) and cognitive functioning (DSST, except SFA). Additionally, they were associated with the SPRINT-BASEed frailty index (cross-sectionally and longitudinally) and Fried’s frailty index (longitudinally). The strongest effect sizes in cross-sectional and longitudinal analyses were found for ALI with respect to diagnosed T2D, MetS, LS7, MI and SPRINT-BASEed frailty index. This presumably results from the way it is calculated, as many of the variables included in the ALI variable are closely related to health and are, in some instances, also part of the diagnostic criteria for T2D, MetS, LS7, and SCORE2. However, sensitivity analyses with modified ALI versions that excluded all variables that were also part of the outcomes in question (MetS, T2D, LS7, SCORE2) remained in almost all cases statistically significant (Supplementary Data 14–16). This increases confidence that ALI is indeed a robust marker for biological age.

Subsequently, we investigated a potential use-case of the markers in a clinical context by predicting incident cases of impairment at follow-up by markers assessed on average 7.4 years earlier at baseline. ALI, GrimAge DNAmAA and DunedinPACE improved our basic prediction model in its prediction of incidence of impairment by up to 24 percentage points (Fig. 4). Although this increase in accuracy of the prediction model is expected to be less substantial when the markers are added to more comprehensive and specific prediction models, these results nevertheless illustrate that the markers hold biological information that can substantially improve the prediction of impairment years before it becomes clinically apparent. To estimate how the predictive value of the markers of aging would change in comparison to more comprehensive clinical prediction models, we calculated a sensitivity analysis that investigated the value added by the markers of aging to an extended clinical model (age, sex, BMI, smoking, and alcohol) (Supplementary Data 19). Although this did not lead to substantial changes in most marker’s interpretation, the added value of ALI with respect to the AUC of the prediction models for MetS, T2D, and LS7 was reduced by up to 17 percentage points compared to our original analysis. This suggests that future studies need to compare individual markers of aging with more comprehensive and specific clinical prediction models to fully understand how the markers of aging can contribute to incident case prediction in a clinical setting. Generally, we found more statistically significant results and stronger effect sizes in the longitudinal analyses compared to the cross-sectional results. This was observable especially for DunedinPACE, Allostatic Load and the psychological aging markers. Markers of aging are expected to depict the underlying aging processes, which are anticipated to happen prior to clinical manifestations. Therefore, it is expected that an accelerated aging process that did not (yet) exceed the individual mechanisms of resilience and therefore did not manifest through clinical phenotypes would be trackable through markers. The results presented here suggest that the respective markers appear to recognize an acceleration in the underlying aging process that was (at least at baseline) not advanced enough or was still compensated by the physiological and cognitive coping mechanisms to not result in clinically observable phenotypes. However, at follow-up examination, the acceleration in the biological aging process, which was already picked up at baseline by the respective markers, potentially resulted in clinical manifestations.

Another main finding of our study is the in part large differences in effect sizes of associations with the examined outcome variables, which suggests that the analyzed markers capture distinct aspects of the biological aging processes^9,17,18,25^. This interpretation of our findings is particularly plausible due to the different concepts of biological age that underlie the examined markers. For example, the first-generation epigenetic clocks aim at the prediction of chronological age and the residuals of a regression of chronological on epigenetic age are used to derive the analyzed marker (DNAmAA)^18,31,32^. Second- and third-generation epigenetic clocks, in contrast, are trained on more complex measures of biological aging calculated from several individual variables^14,33,34^, and DunedinPACE captures the rate of aging comparable to a speedometer^14,70^. Other markers, such as the psychological aging markers, rely on the subjective self-assessment of the participants. Composite markers, like Allostatic Load and BioAge, on the other hand, incorporate information from numerous systems to quantify aging, while other measures, such as telomere length and BrainAge, aim at the quantification of age-related biological changes. These differences result in unique strengths and weaknesses of each marker, which in turn define how they could potentially be used in the scientific and clinical context.

As sex-specific differences in the aging process are well known and to be in line with recommendations for the validation of markers of aging^69^, sex-stratified analyses were presented in the Supplementary Material of this manuscript. In some cases, statistically significant findings that were observable for analyses including the whole study sample did not reach statistical significance in the sex-stratified subgroup analyses. This most likely results from the smaller sample sizes of the respective subgroups. On the other hand, in some cases, it can be seen that the association observed in the whole sample is driven mostly by one of the sex-stratified subgroups. For example, the cross-sectional association between ALI and DCSI (logistic regression, Supplementary Data 6) observed for the whole study sample (OR = 1.4, *p* = 0.00001) seems to be mostly driven by men (OR = 1.5, *p* = 0.00005) compared to the subgroup of women (OR = 1.3, *p* = 0.02) in which the association does not reach the Bonferroni adjusted significance level (Supplementary Data 6). Due to the large number of markers and outcomes, as with the main analyses, we refrained from a detailed discussion of all findings from these subgroup analyses.

Our results are indicative that these markers are indeed capable of depicting underlying aging processes and do so with a higher sensitivity than clinical aging measures. Thereby, they could potentially be used to identify participants who are especially prone to future impairment due to an acceleration in one of the biological aging processes long before they show this decline clinically. Furthermore, we want to note that in this study, the markers of aging are analyzed separately with respect to their ability to predict incident cases in the outcome variables. This neglects the possibility of complementary and synergistic effects when combining markers from different domains. In addition, while the results indicate the promising potential of these markers as screening parameters, the study design does not allow to conclude on any clinical recommendations. Further studies that examine the added value of including these markers in screening processes, ideally in a randomized controlled design, with a meaningful endpoint, are needed. While screening programs are often evaluated with mortality as endpoint, in the aging context, it might be important to analyze other variables that are specific to the outcome of interest and are associated with quality of life and overall health. For example, a reduction of diabetes-associated complications might provide substantial benefit for individuals even if the overall lifespan could not be extended. Therefore, future studies with a focus on adverse outcome prevention are needed to further explore the possibility of utilizing these markers of aging as possible screening markers in a clinical context.

In a previous study by Kuo and colleagues, change in epigenetic age over time was associated with mortality^71^. As a sensitivity analysis, we calculated prediction models investigating the change in the two most promising markers of aging, ALI and DunedinPACE, between T0 and T1 (Supplementary Data 20). Interestingly, DunedinPACE at T0 showed better results compared to the longitudinal change in DunedinPACE, which only remained nominally statistically significant in the unadjusted logistic regression model predicting MI. On the other hand, the longitudinal difference in ALI showed stronger effect sizes when predicting falls, T2D, and MetS, but generally less strong or overall, not statistically significant associations for all other outcome variables. One limitation of our study in this context is that the second timepoint for assessing the longitudinal difference was also used to assess incident cases. Future studies with a third timepoint for incident cases assessment might be needed to evaluate the potential of the aging rate to predict outcomes other than mortality.

We want to point out several limitations to this study. First, the participants of this study are above-average health^26^. Consequently, a comparatively low prevalence of impairment in the functional and cognitive assessments was observed, which could lead to an underestimation of the true effect sizes, and the generalizability of our results to the underlying source population might be limited. Second, the covariates used in the fully adjusted regression models were chosen because of their frequent use in other studies in the field, as well as their known or suspected association with the independent and dependent variables in our regression models. An individual covariate selection for each marker-outcome-combination was outside the scope of this investigation and would also have compromised comparability of effect sizes between markers within this study. Future studies with a stronger focus on causal associations and the mechanistic relationship between markers and outcomes are needed to deepen our understanding. Third, as with any study that reports on a large number of individual statistical tests, multiple testing is an issue. Here, we adjusted the *p*-values using the Bonferroni correction. While this correction is robust, it also comes with the risk of missing true effects due to its conservative approach. Therefore, it is possible that true associations did not reach statistical significance in our study. Finally, the comparatively small age range in BASE-II might be a reason for the weak correlation between some markers and chronological age, and limits our conclusions to this age group. Future longitudinal studies with a wider age range and repeated marker measurements are needed to further improve our understanding of longitudinal associations between the markers investigated here and clinical aging phenotypes.

Strengths of these analyses include the comparative analysis of a large number of markers derived from a wide variety of aging domains, including epigenetics, proteomics, telomeres, composite markers, psychological markers, SkinAge, and BrainAge. The availability of these variables, in addition to numerous age-associated outcomes in a large longitudinal sample, allowed a comprehensive comparison of these markers cross-sectionally as well as over time. We provide information on the distinct abilities of these markers, which can inform future studies and sharpen their profiles. As the measurement of numerous markers is costly, these analyses allow a targeted selection of markers that can be used in future studies based on their strength of association with the specific aging domain of interest.

## Conclusion

Our comparative analyses of 16 markers of aging in the context of a wide range of age-associated outcome variables highlight the distinction between the investigated domains of aging. Interestingly, markers were more frequently and also more strongly associated with outcomes on average 7.4 years after their assessment compared to cross-sectional analyses, suggesting their sensitivity to biological aging processes that became clinically apparent only much later during the follow-up examination. This finding underscores the potential of these markers for early risk stratification. Namely, ALI and DunedinPACE substantially contributed to the prediction of incident impairment at follow-up examination.

## Supplementary information

Supplementary Material

Description of Additional Supplementary Files

Supplementary Data 1-20

Supplementary Data 21

## Data Availability

Source data for Figs. 1–4 are provided as Supplementary Data 5, 9, 18, and 21. Due to concerns for participants’ privacy as well as data protection regulations, BASE-II raw data cannot be made publicly available. Interested investigators are invited to contact the scientific coordinator of BASE-II, Ludmila Müller (lmueller@mpib-berlin.mpg.de), to obtain raw data access for project-specific analyses under an individually negotiated data use agreement. Data requests will be addressed in a timely manner, typically within four weeks of receipt. Additional information can be found on the BASE-II website: https://www.base2.mpg.de/7549/data-documentation.
