# Supplementary material for: Comprehensive cross-sectional and longitudinal comparison of sixteen markers of biological aging from the Berlin Aging Study II
Source: Commun Med (Lond). 2026 Mar 27;6:168. doi: 10.1038/s43856-025-01233-7 (PMC13031708; doi:10.1038/s43856-025-01233-7)
Supplement: Supplementary file 2 — Description of Additional Supplementary Files [file 43856_2025_1233_MOESM2_ESM.docx]

**Description of Additional Supplementary Files**

Supplementary Data 1: Descriptive statistics of variables analyzed in this study at baseline stratified on whether the participants were followed-up on average 7.4 years later.

Supplementary Data 2: Despcriptive statistics of the variables available in the original, non-imputed dataset. Only participants that were assessed at baseline (T0) and follow-up (T1) are included in this table.

Supplementary Data 3: Contiuously scaled variables were dichotomized based on pre-defined cut-offs wherever possible.

Supplementary Data 4: Descriptive statistics of dichotomized continuously scaled variables of the first imputed dataset (n=1,083). McNemar test was used to assess statistical signficance between assessments.

Supplementary Data 5: Cross-sectional linear regression analyses of outcome variables on biomarkers in 1,083 BASE-II participants. BrainAge was only available for a smaller subgroup (n=255). All variables analyzed were assessed at baseline (T0). Model 1: unadjusted; Model 2: age, sex (if applicable); Model 3: Model 2 + PC1, PC2, PC3, PC4, alcohol intake, smoking, BMI.

Supplementary Data 6: Logistic regression analyses of binary outcome variables on biomarkers of aging in 1,083 participants. BrainAge was available for a smaller subgroup only (n=255). Model 1: unadjusted; Model 2: age, sex (if applicable); Model 3: Model 2 + PC1, PC2, PC3, PC4, alcohol intake, smoking, BMI.

Supplementary Data 7: Cross-sectional linear regression analyses of outcome variables on biomarkers in 255 BASE-II participants who provided information about BrainAge. All variables analyzed were assessed at baseline (T0). Model 1: unadjusted; Model 2: age, sex; Model 3: Model 2 + PC1, PC2, PC3, PC4, alcohol intake, smoking, BMI.

Supplementary Data 8: Cross-sectional logistic regression analyses of binary outcome variables on biomarkers of aging in the subgroup of 255 participants who provided information about their BrainAge. All variables are assessed at baseline (T0). Model 1: unadjusted; Model 2: age, sex; Model 3: Model 2 + PC1, PC2, PC3, PC4, alcohol intake, smoking, BMI.

Supplementary Data 9: Longitudinal linear regression analyses of outcome variables at T1 on markers at T0 in 1,083 BASE-II participants. BrainAge was only available for a smaller subgroup (n=255). All covariables were assessed at baseline (T0). Model 1: unadjusted; Model 2: outcome variable at T0, age, sex (if applicable); Model 3: Model 2 + PC1, PC2, PC3, PC4, alcohol intake, smoking, BMI.

Supplementary Data 10: Longitudinal logistic regression analyses of binary outcome variables at T1 on biomarkers of aging at T0 in 1,083 participants. BrainAge was available for a smaller subgroup only (n=255). Covariates were all assessed at T0. Model 1: unadjusted; Model 2: outcome variable at T0, age, sex (if applicable); Model 3: Model 2 + PC1, PC2, PC3, PC4, alcohol intake, smoking, BMI.

Supplementary Data 11: Longitudinal linear regression analyses of outcome variables at T1 on biomarkers at T0 in 255 BASE-II participants who provided informatio about their Brain Age. All covariables were assessed at baseline (T0). Model 1: unadjusted; Model 2: outcome variable at T0, age, sex; Model 3: Model 2 + PC1, PC2, PC3, PC4, alcohol intake, smoking, BMI.

Supplementary Data 12: Longitudinal logistic regression analyses of binary outcome variables at T1 on biomarkers of aging at T0 in the subgroup of 255 participants who provided information about their BrainAge. All covariables are assessed at baseline (T0). Model 1: unadjusted; Model 2: outcome variable at T0, age, sex; Model 3: Model 2 + PC1, PC2, PC3, PC4, alcohol intake, smoking, BMI.

Supplementary Data 13 : Longitudinal linear regression analyses of difference in outcome variables between T0 and T1 on markers of aging at T0 in 1,083 BASE-II participants. BrainAge was only available for a smaller subgroup (n=255). All covariables were assessed at baseline (T0). Model 1: unadjusted; Model 2: outcome variable at T0, age, sex (if applicable); Model 3: Model 2 + PC1, PC2, PC3, PC4, alcohol intake, smoking, BMI.

Supplementary Data 14: Sensitivity analyses of cross-sectional and longitudinal linear regression analyses of the association between cardiovascular health scores and modified version of ALI. As variables overlap between ALI and SCORE2 and LS7, we calculated ageing marker versions without the respective variables. The respectivly modfied versions of ALI therefore do not contain variables which are also part of SCORE2 or LS7. For easier comparison with the original version of the markers, the respective results are shown here again (identical to Supplementary Data 5). All variables analyzed were assessed at baseline (T0). Model 1: unadjusted; Model 2: age, sex (if applicable); Model 3: Model 2 + PC1, PC2, PC3, PC4, alcohol intake, smoking, BMI.

Supplementary Data 15: Sensitivity analyses of cross-sectional and longitudinal logistic regression analyses of the association between cardiovascular health scores, MetS and T2D and modified version of ALI. As variables overlap between ALI and SCORE2, LS7, T2D, and MetS, as a sensitivity analyses we calculated ageing marker versions without the respective variables. The respectivly modfied versions of ALI atherefore do not contain variables which are also part of respective outcome variables. For easier comparison with the original version of the markers, the respective results are shown here again (identical to Supplementary Data 5). All variables analyzed were assessed at baseline (T0). Model 1: unadjusted; Model 2: age, sex (if applicable); Model 3: Model 2 + PC1, PC2, PC3, PC4, alcohol intake, smoking, BMI.

Supplementary Data 16: Sensitivity analyses of logistic regression analyses of incident cases at T1 on biomarkers at T0 in the individual subgroup of participants without prevalent cases of outcome variables at T0. ALI was calculated without variables that were part of SCORE2, LS7, MetS, T2D. Statistical significance between the minimal prediction model (age and sex) and the extended prediction model (age, sex, and biomarker) was assessed by likelihood ratio test. Statistically significant p-values (p<0.0001) and AUC differences of 5% or more are higlighted in grey. Model 1: unadjusted, Model 2: age and sex (if applicable). The minimal predicition model used for comparison to Model 2 consist of age and sex. Note: St. = Standardized; OR = Odds Ratio; AUC = Area Under the Curve

Supplementary Data 17: Sensitivity analysis of logistic regression analyses of incident cases at T1 on GrimAge DNAmAA and GrimAge2 DNAmAA at T0 in the individual subgroup of participants without prevalent cases of outcome variables at T0. As the definition of impairment followed the multiple imputation process to gurantee internal consistency within all imputed datasets, the number of prevalent cases at T0 varies which results in differing sample sizes for the analyses performed here. Statistical significance between the minimal prediction model (age and sex) and the extended prediction model (age, sex, and biomarker) was assessed by likelihood ratio test. Statistically significant p-values (p<0.0001) and AUC differences of 5% or more are higlighted in grey. Model 1: unadjusted, Model 2: age and sex (if applicable). The minimal predicition model used for comparison to Model 2 consist of age and sex. Note: St. = Standardized; OR = Odds Ratio; AUC = Area Under the Curve.

Supplementary Data 18: Logistic regression analyses of incident cases at T1 on biomarkers at T0 in the individual subgroup of participants without prevalent cases of outcome variables at T0. As the definition of impairment followed the multiple imputation process to gurantee internal consistency within all imputed datasets, the number of prevalent cases at T0 varies which results in differing sample sizes for the analyses performed here. Statistical significance between the minimal prediction model (age and sex) and the extended prediction model (age, sex, and biomarker) was assessed by likelihood ratio test. Statistically significant p-values (p<0.0001) and AUC differences of 5% or more are higlighted in grey. Model 1: unadjusted, Model 2: age and sex (if applicable). The minimal predicition model used for comparison to Model 2 consist of age and sex. Note: St. = Standardized; OR = Odds Ratio; AUC = Area Under the Curve

Supplementary Data 19: Sensitivity analyses of prediction models comparing an extended clinical model (age, sex, BMI, smoking, alcohol) with the markers of ageing as well as the difference in model performance to the extended clinical model with the included marker of ageing (age, sex, BMI, smoking, alcohol, marker of ageing). Statistical significance between the extended prediction model and the extended prediction model + marker of ageing was assessed by likelihood ratio test. Model 1: unadjusted, Model 2: age, sex, BMI, smoking, and alcohol. Note: St. = Standardized; OR = Odds Ratio; AUC = Area Under the Curve

Supplementary Data 20: Logistic regression analyses of incident cases at T1 on on change in ALI and DunedinPACE between T0 and T1 in the individual subgroup of participants without prevalent cases of outcome variables at T0. As the definition of impairment followed the multiple imputation process to guarantee internal consistency within all imputed datasets, the number of prevalent cases at T0 varies which results in differing sample sizes for the analyses performed here. Statistical significance between the minimal prediction model (age and sex) and the extended prediction model (follow-up time, age, sex, and biomarker) was assessed by likelihood ratio test. Statistically significant p-values (p<0.0001) and AUC differences of 5% or more are higlighted in grey. Model 1: unadjusted, Model 2: age and sex. The minimal predicition model used for comparison to Model 2 consist of age and sex. Note: St. = Standardized; OR = Odds Ratio; AUC = Area Under the Curve

Supplementary Data 21: Source data behind Figure 4.
